# RNA-seq data of invasive ductal carcinoma and adjacent normal tissues from a Korean patient with breast cancer

**DOI:** 10.1016/j.dib.2018.03.079

**Published:** 2018-03-21

**Authors:** Ji Hyung Hong, Yoon Ho Ko, Keunsoo Kang

**Affiliations:** a Division of Oncology, Department of Internal Medicine, College of Medicine, The Catholic University of Korea, Seoul, Republic of Korea; b Cancer Research Institute, College of Medicine, The Catholic University of Korea, Seoul, Republic of Korea; c Department of Microbiology, College of Natural Sciences, Dankook University, Cheonan 31116, Republic of Korea

**Keywords:** Breast cancer, Luminal B subtype, Invasive ductal carcinoma, RNA-seq, Korean

## Abstract

Invasive ductal carcinoma is the most common type of breast cancer. Here, we provide a whole transcriptome shotgun sequencing (called RNA-seq) dataset conducted with ten samples of invasive ductal carcinoma tissue and three samples of adjacent normal tissue from a single Korean breast cancer patient (luminal B subtype). Differentially expressed genes (DEGs) were identified with a false discovery rate (FDR)-adjusted *p*-value of 0.05. Gene ontology analysis identified several key pathways, including lymphocyte activation. A list of differentially expressed genes is provided. The raw data was uploaded to the sequence read archive (SRA) database and the BioProject ID is PRJNA432903.

**Specifications Table**

**Table:** 

Subject area	*Biology*
More specific subject area	*NGS, Transcriptomics, Cancer biology*
Type of data	*Transcriptome data*
How data was acquired	*High-throughput sequencing using Illumina HiSeq2500*
Data format	*Raw (fastq)*
Experimental factors	*Breast cancer (invasive ductal carcinoma; luminal B subtype) and adjacent normal tissues*
Experimental features	*Poly(A) RNA was purified from 1 g total RNA from each sample, and cDNA was synthesized using SuperScript II (Invitrogen). Sequencing libraries were prepared using the TruSeq RNA Library preparation kit (Illumina)*
Data source location	*Seoul, Republic of Korea*
Data accessibility	*Raw data can be accessed at NCBI SRA (BioProject ID: PRJNA432903) (*https://www.ncbi.nlm.nih.gov/bioproject/PRJNA432903*).*

**Value of the data**

• This RNA-seq data provides a deep sequencing of ten samples of invasive ductal carcinoma tissue and three samples of adjacent normal tissue from a Korean breast cancer patient (luminal B subtype)
• The heterogeneous expression data from spatially distinct tumor samples can be used for various evaluation purposes.
• Gene ontology analysis revealed that lymphocyte activation and PPAR signaling pathway are significantly up- and down-regulated pathways, respectively, in breast cancer tissue compared to adjacent normal tissue.

## 1 Data

Total RNA was extracted from ten samples of cancer tissue (invasive ductal carcinoma; luminal B subtype) and three samples of adjacent normal tissue from a Korean patient with breast cancer. RNA-seq was performed to profile transcriptomes of breast cancer and normal samples. Differentially expressed genes were identified with an FDR-adjusted *p*-value cutoff of 0.05. Gene ontology analysis indicated that several pathways are associated with the onset or progression of breast cancer.

## 2 Experimental design, materials and methods

### 2.1 RNA-seq

One tissue sample of invasive ductal carcinoma (luminal B subtype) from breast tissue and a corresponding adjacent normal tissue were biopsied from a Korean woman with informed consent. This study was approved by the institutional review board of Catholic Medical Center (approval no. UC17TISI0015). The tumor and adjacent normal tissues were divided into ten and three samples, respectively. Poly(A) RNA was purified from 1 g total RNA from each sample, and cDNA was synthesized using SuperScript II (Invitrogen). Sequencing libraries were prepared using the TruSeq RNA Library preparation kit (Illumina) and sequenced using HiSeq. 2500 (Illumina).

### 2.2 RNA-seq analysis

Sequenced reads were trimmed using Trim Galore (version 0.4.2; https://www.bioinformatics.babraham.ac.uk/projects/trim_galore/) with Cutadapt (version 1.1.2) [1]. Trimmed reads were mapped to the reference human genome (hg38) using STAR (version 2.5.2b) [2]. The PCR-duplicate removal of mapped reads was performed using Sambamba (version 0.6.5) [3]. The quality of RNA-seq data was determined using RSeQC (version 2.6.4) with the transcript integrity number (TIN) score (Table 1) [4]. The abundances of RefSeq genes were estimated using Cufflinks with the Cuffnorm function (version 2.2.1) (Supplementary Table 1) [5].

**Table 1:** RNA quality was measured using the transcript integrity number (TIN) score.

Cancer	TIN score (median)	Normal	TIN score (median)
C0	79.6	N1	79.3
C1	80.1	N2	65.6
C2	79.7	N3	67.8
**C3**	**59.6**		
C4	79.1		
C5	79.8		
C6	80.6		
C7	78.1		
C8	80.1		
C9	80.1		

### 2.3 Identification of differentially expressed genes

**Fig. 1:**
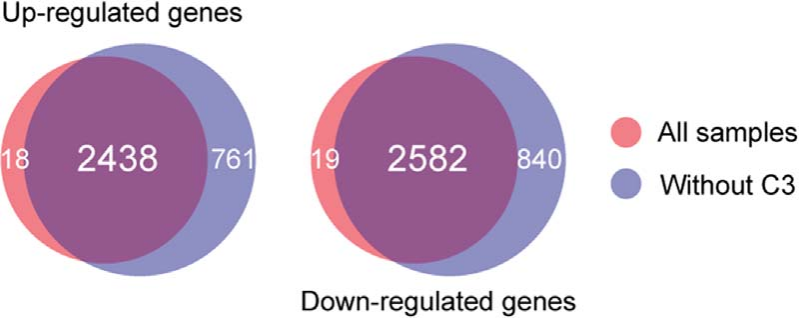
Comparison of differentially expressed genes. Venn diagrams show the number of common and unique DEGs between different DEG analyses. DEG analyses were performed with or without the C3 sample.

Differentially expressed genes (DEGs) between cancer and normal samples were identified using Cufflinks with the Cuffdiff function (version 2.2.1) [5]. DEGs were defined as the genes with FDR-adjusted *p*-values <0.05. A total of 2456 up-regulated and 2601 down-regulated genes were identified in cancer samples compared to adjacent normal samples (Supplementary Table 2). When the low-quality RNA-seq data (C3) was excluded for DEG analysis, a total of 3199 up-regulated and 3422 down-regulated genes were identified as DEGs (Fig. 1 and Supplementary Table 3).

### 2.4 Gene ontology analysis

**Fig. 2:**
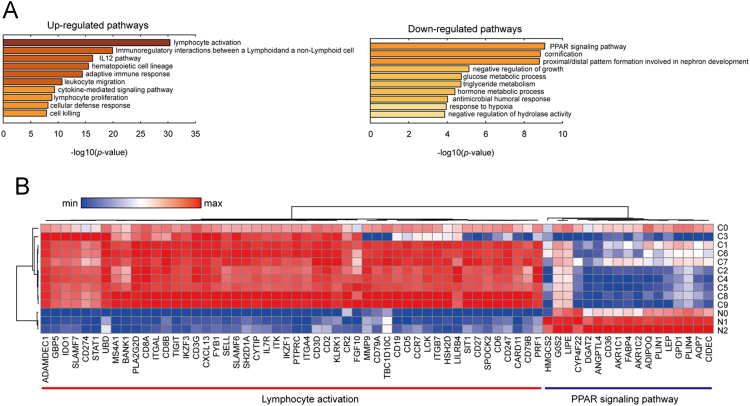
Gene ontology analysis showing altered pathways in breast cancer tissue compared to adjacent normal tissue. (A) Pathways significantly (*p*-value <0.05) associated with up- and down-regulated genes are shown. (B) The heatmap shows relative expression levels of the genes that are involved in lymphocyte activation and PPAR signaling pathway.

Gene ontology (GO) analysis was performed to identify key pathways regarding the DEGs that were identified without the C3 sample. The top 100 up-regulated (or down-regulated) DEGs that were highly expressed (> 10 average FPKM) were analyzed using Metascape (http://metascape.org) [6]. The GO analysis revealed that the majority of up-regulated genes were significantly associated with lymphocyte activation and that some down-regulated genes were involved in PPAR signaling pathway (Fig. 2).
